# Impact of the Growing Healthy mHealth Program on Maternal Feeding Practices, Infant Food Preferences, and Satiety Responsiveness: Quasi-Experimental Study

**DOI:** 10.2196/mhealth.9303

**Published:** 2018-04-25

**Authors:** Catherine Georgina Russell, Elizabeth Denney-Wilson, Rachel A Laws, Gavin Abbott, Miaobing Zheng, Sharyn J Lymer, Sarah Taki, Eloise-Kate V Litterbach, Kok-Leong Ong, Karen J Campbell

**Affiliations:** ^1^ Centre for Advanced Sensory Science School of Exercise and Nutrition Sciences, Faculty of Health Deakin University Burwood Australia; ^2^ Centre for Obesity Management and Prevention Research Excellence in Primary Health Care Sydney Australia; ^3^ Sydney Nursing School The University of Sydney Sydney Australia; ^4^ Sydney Local Health District Sydney Australia; ^5^ Institute for Physical Activity and Nutrition School of Exercise and Nutrition Sciences Deakin University Geelong Australia; ^6^ The Boden Institute of Obesity Nutrition Exercise & Eating Disorders Charles Perkins Centre University of Sydney Sydney Australia; ^7^ Health Promotion Unit Sydney Local Health District and University of Sydney Sydney Australia; ^8^ Department of Accounting and Data Analytics, La Trobe Business School College of Arts, Social Sciences and Commerce La Trobe University Melbourne Australia

**Keywords:** mHealth, obesity, infant, parents, food preferences, appetite, pediatric obesity, feeding behavior, overweight, eating, health promotion

## Abstract

**Background:**

Infancy is an important life stage for obesity prevention efforts. Parents’ infant feeding practices influence the development of infants’ food preferences and eating behaviors and subsequently diet and weight. Mobile health (mHealth) may provide a feasible medium through which to deliver programs to promote healthy infant feeding as it allows low cost and easy access to tailored content.

**Objective:**

The objective of this study was to describe the effects of an mHealth intervention on parental feeding practices, infant food preferences, and infant satiety responsiveness.

**Methods:**

A quasi-experimental study was conducted with an mHealth intervention group (Growing Healthy) and a nonrandomized comparison group (“Baby's First Food"). The intervention group received access to a free app with age-appropriate push notifications, a website, and an online forum that provided them with evidence-based advice on infant feeding for healthy growth from birth until 9 months of age. Behavior change techniques were selected using the Behaviour Change Wheel framework. Participants in both groups completed three Web-based surveys, first when their infants were less than 3 months old (baseline, T1), then at 6 months (time 2, T2), and 9 months of age (time 3, T3). Surveys included questions on infant feeding practices and beliefs (Infant Feeding Questionnaire, IFQ), satiety responsiveness (Baby Eating Behaviour Questionnaire), and infant’s food exposure and liking. Multivariate linear regression models, estimated using maximum likelihood with bootstrapped standard errors, were fitted to compare continuous outcomes between the intervention groups, with adjustment for relevant covariates. Multivariate logistic regression adjusting for the same covariates was performed for categorical outcomes.

**Results:**

A total of 645 parents (Growing Healthy: n=301, Baby's First Food: n=344) met the eligibility criteria and were included in the study, reducing to a sample size of 546 (Growing Healthy: n=234, Baby's First Food: n=312) at T2 and a sample size of 518 (Growing Healthy: n=225, Baby's First Food: n=293) at T3. There were approximately equal numbers of boy and girl infants, and infants were aged less than 3 months at baseline (Growing Healthy: mean 7.0, SD 3.7 weeks; Baby's First Food: mean 7.9, SD 3.8 weeks), with Growing Healthy infants being slightly younger than Baby's First Food infants (*P*=.001). All but one (IFQ subscale “concerns about infant overeating or becoming overweight” at T2) of the measured outcomes did not differ between Growing Healthy and Baby's First Food.

**Conclusions:**

Although mHealth can be effective in promoting some health behaviors and offers many advantages in health promotion, the results of this study suggest that design and delivery characteristics needed to maximize the impact of mHealth interventions on infant feeding are uncertain. The sensitivity of available measurement tools and differences in baseline characteristics of participants may have also affected the results.

## Introduction

### Context

Childhood obesity is a strong risk factor for adult overweight or obesity [1,2] and is associated with numerous medical, psychosocial, and economic costs [3-5]. Primary prevention is therefore an important public health priority, particularly given the high prevalence in numerous societies [6,7], including Australia, where approximately one-quarter of children are overweight or obese [8]. Rapid growth in infancy has a strong positive association with being overweight in childhood [9,10] and is therefore an important target for prevention efforts. The World Health Organization’s (WHO) Commission on Ending Childhood Obesity [11] notes that to reverse worldwide trends in childhood obesity, approaches for addressing the complex range of risk factors in a range of population groups is needed [12]. The World Health Organization urges “Member States to develop national responses, strategies and plans to end infant, child and adolescent obesity” [13]. One modifiable risk factor is parental feeding practices that encompass those food- or eating-specific behaviors or strategies that parents use to influence children’s or infants’ eating.

### The Importance of Parental Feeding Practices and Cognitions to Infant Eating and Weight

Parental feeding practices affect infants’ and children’s food intakes, as well as the development of their food preferences and eating behaviors (such as responding to the hunger and satiety cues) [14-16]. They are important intervention targets because they influence healthy eating and weight in both the short and long term. Feeding practices likely to promote healthy eating and weight outcomes for children or infants typically incorporate responsive feeding (ie, recognizing and responding to an infant’s hunger and satiety cues and needs in appropriate ways) with a flexible feeding or mealtime structure (ie, providing consistency in what, when, and how food is provided) [17-20]. Conversely, nonresponsive feeding practices include those that are controlling or pressuring, which can elevate a child’s risk of weight gain [21]. These feeding practices are characterized by a higher disregard for an infant or child’s hunger and satiety cues and attempts to promote either higher or lower consumption of particular foods through, for example, restricting children’s access to or consumption of particular foods. These types of feeding practices can disrupt an infant’s or child’s ability to effectively self-regulate their caloric intake, leading to excess calorie intake and weight gain [22]. However, there are still many gaps in our understanding of such relationships.

Parental cognitions such as concerns about an infant eating too much, or that a child is at risk of becoming overweight, may affect their feeding practices. Parents who are concerned about their infant or child being or becoming underweight or not gaining enough weight are more likely to use pressuring feeding practices to promote greater consumption [23,24]. Conversely, parents who are concerned about their child being or becoming overweight are more likely to restrict access to foods [25]. These concerns and perceptions may arise partly in reaction to the characteristics of the infant (eg, birth weight, speed of eating, or gender), as well as those of the caregiver such as the mother’s education level and her own weight status [26,27].

### The Importance of Food Preferences to Eating and Weight

Also significant in the development of weight status are children’s emerging food preferences: these are an important determinant of whether children consume particular foods or avoid them [28,29], especially vegetables [30]. Repeated exposure is a core determinant of food liking in children and infants, with higher exposure typically linked to higher liking [31]. The types of foods that infants and children are exposed to will have a lasting influence on their developing food preferences and whether they are likely to consume healthy diets in the future [32,33]. Consequently, it is important that parents repeatedly expose infants to core foods that are associated with healthy weight gain and growth and avoid noncore foods that are associated with poorer diets and health. Parental feeding practices, parental feeding cognitions, and children’s food preferences are three potentially modifiable domains that may influence child weight outcomes.

### Approaches to Promoting Healthy Feeding and Eating in Infancy

Despite the range of studies now indicating that particular parental feeding practices and associated cognitions in early stages of children’s lives are important for children’s healthy weight gain, there are still substantial gaps in our understanding of the most effective approaches for helping parents to achieve this [34]. Available evidence suggests that by providing guidance to parents, some, but not all, feeding practices and beliefs can be shifted to healthier patterns [34]. Evidence for this comes from multicomponent behavioral interventions that broadly aimed to increase parents’ knowledge and skills about infant feeding in face-to-face individual or group settings [34]. Due to the delivery mode, these interventions are necessarily resource intensive and are therefore less able to be provided to a wide range of potential participants. Mobile health (mHealth), in contrast, provides the advantages of flexibility in how and when information is accessed by participants at relatively low cost of use and dissemination. Given the high penetration of mobile phones [35], mHealth interventions may be more likely to be used by a wide range of sociodemographic groups, including those typically hard to reach with face-to-face interventions such as parents of lower socioeconomic position.

mHealth programs also offer advantages over traditional approaches in the types of behavior change approaches that can be employed, which may enhance intervention effectiveness. For instance, content can be readily tailored to participants’ individual needs (eg, infant’s age and whether breast- or formula-feeding), and because they offer programming flexibility, numerous behavior change techniques (BCTs) can be readily utilized (eg, video demonstrations and feedback on behaviors) or features be incorporated (eg, prompts). For these reasons, mHealth approaches to health promotion provide an attractive medium through which interventions could be delivered to time-poor groups such as new parents. Available evidence from the wider mHealth field suggests that mHealth interventions are more effective in promoting some health behaviors than others [36-38]. A recent review found that their capacity to influence infant or child eating or weight through parents as an agent of change is, however, uncertain [39].

To this end, this paper reports on data from the *Growing Healthy* program, an mHealth intervention that aimed to promote infant feeding practices consistent with national guidelines from birth until 9 months of age [40]. The purpose of this paper is to describe whether parents participating in this mHealth intervention were more likely than those in a nonrandomized comparison group to (1) use feeding practices associated with healthy weight outcomes in infants or children; (2) be more likely to expose infants to core, as opposed to noncore foods, (3) have infants who like more core foods and like fewer noncore foods, and (4) have infants who are better at responding to internal hunger and fullness cues.

## Methods

### Overview

Details of the study design are reported elsewhere [40]. In brief, the study used a quasi-experimental study design with an mHealth intervention group and a nonrandomized comparison group. Ethics approval was provided by Deakin University and University of Technology Sydney.

### Study Participants

Eligibility criteria for participation in the intervention group (Growing Healthy) included pregnant (30+ weeks gestation) or parent or main caregiver of an infant aged under 3 months, owned any type of mobile phone, spoke and read English, aged 18 years or older, and lived in Australia. Participants were recruited three ways: via their primary health care providers in socioeconomically disadvantaged communities in two Australian states (New South Wales and Victoria), face-to-face by researchers, and through Web advertising. Enrollment to the study included completion of a Web-based screening form, a consent form, and a baseline survey. A concurrent nonrandomized comparison group (Baby's First Food) was recruited via Web forums, social networking sites, and blogs and received usual care that involves regular face-to-face appointments with a maternal and child nurse to monitor and advise on the infant’s health, growth, and development. Further details can be found in the study by Laws et al [41].

### Intervention: The Growing Healthy Program

The Growing Healthy program consisted of an app, website, and Web-based forum, providing parents with evidence-based advice on infant feeding for healthy growth from birth until 9 months of age. The program aims included promoting healthy infant feeding practices (eg, recognizing and appropriately responding to infant cues of hunger and satiety) and promoting high exposure to fruits and vegetables [40]. Participants received three push notifications via their Growing Healthy app (or via email for those without a mobile phone) for each week of the intervention on infant feeding topics tailored to their infant’s age and feeding mode (whether breast-, formula-, mixed-feeding, alone or in combination with solid-feeding). The push notifications and emails provided links to further related information on the app or website. The app and website contained enriched information delivered in a variety of formats (eg, video, text, and imagery), a range of communication functions (eg, capacity to share with others), and a Web-based forum. Detailed information on the intervention and its development is reported elsewhere [40]. BCTs were selected using the Behaviour Change Wheel (BCW) framework [42] as a guide. That is, the BCW was used to identify determinants of the target behaviors and their alternatives (less desirable behaviors) and to map these to BCTs using the behavior change taxonomy (see [43,44]).

### Data Collection

Participants in Growing Healthy and Baby's First Food completed a baseline survey when their infant was between 2 weeks and 3 months of age, a follow up survey when infants were approximately 6 months of age, and a final survey when infants were approximately 9 months. The baseline survey collected information on sociodemographics, including the child’s age and gender and the mothers’ age, country of birth (Australia or overseas born), relationship status (single or married), employment status (currently employed or unemployed), education level (low: no formal education or high school; medium: certificate or diploma; high: university degree and higher), and annual household income (Aus $≤51,999, 52,000-77,999, 78,000-99,999, ≥100,000). Mothers also self-reported their prepregnancy weight in kilograms and current height in centimeters. Maternal prepregnancy body mass index (BMI) was calculated as prepregnancy weight divided by height squared (kg/m^2^). Feeding mode (exclusively breastfeeding, formula feeding, or mixed feeding) was also collected.

#### Assessment of Parental Feeding Practices and Beliefs

Parental feeding practices and beliefs were collected at all three time points using questions from the Infant Feeding Questionnaire (IFQ) [45]. The IFQ consists of 20 items across the seven dimensions of: concern about infant undereating or becoming underweight (example item: do you worry that your baby is not eating enough?), concern about infant’s hunger (example item: do you put cereal in his bottle so he stays full longer?), awareness of infant’s hunger and satiety cues (example item: my baby knows when he is hungry), concern about infant overeating or becoming overweight (example item: I am worried that my baby would become overweight), feeding infant on a schedule (example item: do you feed your baby at set times?), using food to calm infant’s fussiness (example item: feeding my baby is the best way to stop him being unsettled), and social interaction with the infant during feeding (example item: do you talk or sing to your baby when you feed him?). Behavioral items were measured on a 5-point frequency scale (0=never, 1=rarely, 2=sometimes, 3=often, and 4=always), whereas belief items were measured on the following scale: 0=disagree a lot, 1=disagree a little, 2=no strong feelings either way, 3=agree a little, and 4=agree a lot.

#### Assessment of Infant Satiety Responsiveness

Respondents were also asked about perceptions of their infant’s ability to respond to their internal satiety cues (*satiety responsiveness*), which was measured with three items from the Baby Eating Behaviour Questionnaire (BEBQ, [46]. In total, 6 of the 20 items were not used in all three variants of the survey at each time point, and for consistency, were therefore excluded from analyses.

#### Assessment of Infant Food Exposure and Parental Intentions to Offer Foods

Frequency of food exposure, infant food preference, and parents’ intentions to offer foods again were reported at time 3 (T3) with purpose-developed items. Thirty-two foods were included to provide a range of foods, typically available in the Australian food supply and being characteristic of foods recommended to be consumed in high or low amounts [47]. The 22 core foods were apple, banana, grape, orange, watermelon, mandarin, pear, rockmelon, kiwi, grapefruit, potato, carrot, pumpkin, broccoli, corn, tomato, mushroom, sweet potato, parsnip, eggplant, water, and cow’s milk. The ten noncore foods were juice, other drinks, flavored milk, cakes, sweet biscuits, savory biscuits, chocolate or lollies, salty snacks, pies, and hot chips (French fries). For each food item, parents were asked about the frequency at which they had offered the food (1=never, 2=less than once a month, 3=1-3 times a month, 4=once a week, 5=2-4 times a week, 6=5-6 times a week, and 7=once a day or more) and about their infant’s liking of the food (question: does the child usually like this food? response categories: yes, no, and hasn’t tried this food), as well as their intentions to reoffer foods (question text: will you offer this food again in the next six months? response categories: yes, no, and unsure). Additional questions on whether parents added sugar or salt into foods their infant would eat were also asked (response options: never, sometimes, often, and always).

### Statistical Analysis

For baseline characteristics, group comparisons were made using *t* tests for continuous characteristics and chi-square tests for categorical characteristics. Exploratory factor analysis (promax rotation) was performed on the 14 IFQ at all three time points. At each time point, five main factors were extracted and were consistent with the original IFQ structure [48]. These factors were as follows: (1) concern about infant undereating or becoming underweight (4 items), (2) awareness of infant hunger and satiety cues (3 items), (3) concern about infant overeating or becoming overweight (3 items), (4) feeding infant on a schedule (2 items), and (5) using food to calm infant fussiness (2 items). The scores of these five outcomes were calculated as the sum of the corresponding subitems, with higher scores representing stronger beliefs or more frequent behaviors for each factor. For the *satiety responsiveness* score from the BEBQ, the three items were added. The list of items included in each outcome is provided in Multimedia Appendix 1.

To assess infants’ food exposure, we calculated core and noncore food offering frequency scores and variety scores. Frequency of consumption of each food item was converted to daily equivalent scores (never=0, less than once a month=0.017, 1-3 times a month=0.067, once a week=0.143, 2-4 times a week=0.429, 5-6 times a week=0.786, and once a day or more=1). Adding daily equivalent scores of the 22 core food categories and ten noncore food categories, respectively, generated core and noncore food frequency scores. For the food variety score, frequency of consumption of each item was first coded into a binary variable indicating offered or not offered. Core and noncore food variety scores were created by adding individual binary variables together, resulting in a score ranging from 0 to 22 for core foods and 0 to 10 for noncore foods. Similarly, by adding binary variables (offer again yes or no) of individual foods together, the number of core or noncore foods that parent will offer again was also obtained. For infants who disliked one or more core foods, a score was created for the proportion of disliked core foods that the parent intended to offer again in the next 6 months. For infant food preferences, individual food item preference was coded as either yes or no, with “has not tried” coded to missing. The proportions of core and noncore foods that the infant tasted and disliked, as well as the proportion of disliked core foods the parent intended to offer again, were dichotomized into all versus not all. Questions asking whether parents added sugar or salt to baby foods were combined into a single outcome and dichotomized as never versus some of the time (sometimes or often or always).

Descriptive analyses (ie, means and SDs for continuous variables and percentages for categorical variables) were conducted to compare baseline characteristics between the two groups. Multivariate linear regression models, estimated using maximum likelihood with bootstrapped standard errors, were fitted to compare continuous outcomes between the intervention groups, with adjustment for baseline parental feeding practice and belief variables, and covariates including infant’s age, maternal age, maternal BMI, whether first born, maternal country of birth, and feeding method. These covariates were chosen as they each differed between Growing Healthy and Baby's First Food groups and were associated with at least one outcome variable with *P*<.25, allowing for the inclusion of potentially important confounders. Multivariate logistic regression adjusting for the same aforementioned covariates was performed for categorical outcomes. As noncore food exposure (frequency scores) residuals of linear regression analyses were right skewed, this variable was also analyzed as a dichotomized variable: whether parents offer noncore foods to their infant (never or some). Attrition analysis was performed to examine baseline characteristics of those who remained in the study and those who dropped out. All analyses were conducted in Stata (Release 14; StataCorpLP).

## Results

### Retention and Recruitment

There were 645 eligible participants at baseline (Growing Healthy: 301, Baby's First Food: 344), reducing to a sample size of 546 (Growing Healthy: n=234, Baby's First Food: n=312) at time 2 (T2) and a sample size of 518 (Growing Healthy: n=225, Baby's First Food: n=293) at T3. Thus 82 participants (82/645, 12.7%) dropped out between baseline and T2, and a further 28 participants (making a total of 110 [28/645, 17%]) dropped out between T2 and T3. Most (151/301, 50.3%) of the intervention group was recruited via the Web, 7.7% (23/301) through face-to-face methods, 29.3% (88/301) via practitioners, and the remainder (38/301, 12.7%) via word of mouth. Further details are described in the papers by Laws et al [41,49].

### Study Participants at Baseline

Details of recruitment and retention of study participants are reported elsewhere [41]. As shown in Table 1, baseline infant characteristics between two groups were similar, with exception of infant age and proportion of first-born infants: the mean infant age in Growing Healthy (7.0, SD 3.7, weeks) was younger than those in Baby's First Food (7.9, SD 3.8, weeks), and there was a greater proportion of first-born infants in Growing Healthy (173/301, 57.5%) than in Baby's First Food (133/344, 38.7%). The proportion of boys in both groups was similar (150/301, 49.8% in Growing Healthy and 167/344, 48.5% in Baby's First Food). The distribution of feeding mode also differed between the two groups, with a lower proportion of exclusive breastfeeding mothers in Growing Healthy (196/301, 65.1%) than in Baby's First Food (245/344, 71.2%). For parental characteristics, apart from Growing Healthy mothers being younger (Growing Healthy: mean 30.4, SD 4.7 years vs Baby's First Food: mean 31.2, SD 4.4 years), a lower proportion being Australian born (Growing Healthy: 253/301, 84.1% vs Baby's First Food: 310/344, 90.1%), and a higher proportion coming from middle household income categories (Growing Healthy: 145/301, 56.9% vs Baby's First Food: 138/344, 47.9%), other parental factors such as mother’s smoking status, prepregnancy BMI, parental education, and employment status were not statistically different. The entire sample at baseline (n=645) and 93% (510/546) at T2 completed the survey questions pertaining to parental feeding practices and beliefs (IFQ). Ninety-three percent of participants (480/518) at T3 completed questions relating to dietary exposure, infant food preferences, and intentions to reoffer disliked foods, in addition to the IFQ. Baseline parental IFQ and infant *satiety responsiveness* (BEBQ) scores by intervention groups are shown in Table 2. No significant between-group differences were observed at baseline. Details of the individual items comprising the IFQ factors and the Cronbach alphas for each IFQ factor at baseline, T2 and T3 are in Multimedia Appendix 1.

There were some statistically significant differences between the retained samples and study dropouts with respect to baseline characteristics. Participants who had dropped out by T2 had lower baby birth weight (mean 3321.6, SD 738.4 g vs mean 3485.6, SD 644.3) than the retained T2 sample. Participants who had dropped out by T3 had lower baby birth weight (mean 3350.6, SD 776.3 vs mean 3492.7, SD 624.1), parents perceived them to have an easier or better baby temperament (mean 2.2, SD 0.9 vs mean 2.4, SD 0.8), greater awareness of infant hunger and satiety cues (mean 13.0, SD 2.1 vs mean 12.6, SD 2.0), were less likely to be married (118/127, 92.3% vs 503/518, 97.1%), more likely to have a health care card (28/127, 22.1% vs 73/518, 14.1% ), and less likely to be tertiary educated (48/124, 38.7% vs 263/507, 51.9%) than the retained T3 sample.

### Outcomes at Time 2

Five outcomes relating to parental feeding practice and belief outcomes and one outcome on infant satiety responsiveness scores were examined at T2 when infant mean age was 6 months (26.6 weeks). Comparison of these outcomes by intervention groups is presented in Table 3. Adjusted mean differences between the two groups were not significantly different for any of the outcomes examined with the exception of IFQ subscale “Concerns about infant overeating or becoming overweight,” which was higher in Growing Healthy. The median score for the IFQ subscale “Concerns about infant undereating or becoming underweight” was 7.0 for both groups. Median score of the IFQ subscale “Awareness of infant hunger and satiety cues” was not significantly different between Baby's First Food (14.0) and Growing Healthy (13.0). Both groups had similar median score for the IFQ subscales “Feeding infant on a schedule” (7.0), “Using food to calm infant fussiness” (6.0), and the BEBQ infant “Satiety responsiveness score” (7.0).

**Table 1:** Sociodemographic characteristics of the Baby's First Food and Growing Healthy samples at baseline.

Characteristics			Growing Healthy (n=301)	Baby's First Food (n=344)	*P* value
**Child factors**					
	Age (weeks)		7.0 (3.7)	7.9 (3.8)	.001
	**Gender, n (%)**				
		Boys	150 (49.8)	167 (48.5)	.74
		Girls	151 (50.2)	177 (51.5)	
	First born baby, n (%)		173 (57.5)	133 (38.7)	<.001
**Parental factors**					
	Mother’s age (years)		30.4 (4.7)	31.2 (4.4)	.04
	Mother prepregnancy body mass index (kg/m^2^), mean (SD)		26.6 (5.7)	27.2 (6.8)	.23
	Maternal smoking status (currently smoking), n (%)		18 (6.0)	15 (4.4)	.35
	Maternal country of birth (Australian born), n (%)		253 (84.1)	310 (90.1)	.02
	Relationship status (married), n (%)		289 (96.0)	332 (96.5)	.74
	Health care card (yes), n (%)		48 (16.0)	53 (15.4)	.85
	**Maternal self-rated health, n (%)**				
		Poor or fair	30 (10.0)	28 (8.1)	.51
		Good	116 (38.5)	152 (44.2)	
		Very Good	124 (41.2)	131 (38.1)	
		Excellent	31 (10.3)	33 (9.6)	
	**Maternal education, n (%)**				
		Low	61 (21.1)	56 (16.4)	.29
		Medium	88 (30.5)	115 (33.6)	
		High	140 (48.4)	171 (50.0)	
	**Maternal working status, n (%)**				
		Not working	261 (86.7)	298 (87.1)	.87
		Working	40 (13.3)	44 (12.9)	
	**Paternal education, n (%)**				
		Low	56 (19.4)	64 (19.3)	.56
		Medium	144 (49.8)	153 (46.1)	
		High	89 (30.8)	115 (34.6)	
	**Paternal working status, n (%)**				
		Not working	12 (4.2)	7 (2.1)	.14
		Working	277 (95.8)	324 (97.9)	
	**Annual house income (AUD), n (%)**				
		≤51,999	35 (13.7)	44 (15.3)	.02
		52,000-77,999	79 (31.0)	57 (19.8)	
		78,000-99,999	66 (25.9)	81 (28.1)	
		≥100,000	75 (29.4)	106 (36.8)	
	**Feeding groups, n (%)**				
		Exclusive breastfeeding	196 (65.1)	245 (71.2)	<.001
		Formula feeding	52 (17.3)	48 (14.0)	
		Mixed feeding	53 (17.6)	51 (14.8)	

**Table 2:** Baseline parental feeding practice and beliefs (Infant Feeding Questionnaire) and infant satiety responsiveness (Baby Eating Behaviour Questionnaire) in the Baby's First Food and Growing Healthy samples. IQR: interquartile range.

Baseline (mean infant age: 7.4 weeks)	Baby's First Food (n=344)			Growing Healthy (n=301)		
	n	Mean (SD)	Median (IQR)	n	Mean (SD)	Median (IQR)
Concerns about infant undereating or becoming underweight (4 items, maximum score 20)	343	7.1 (2.8)	6.0 (5.0-8.0)	301	7.3 (2.7)	7.0 (5.0-9.0)
Awareness of infant hunger and satiety cues (3 items, maximum score 15)	344	12.8 (2.1)	13.0 (12.0-15.0)	301	12.6 (2.0)	13.0 (11.0-14.0)
Concerns about infant overeating or becoming overweight (3 items, maximum score 15)	344	5.0 (2.1)	4.0 (3.0-6.0)	301	5.3 (2.0)	5.0 (4.0-7.0)
Feeding infant on a schedule (2 items, maximum score 10)	343	3.8 (1.8)	3.0 (2.0-5.0)	301	3.8 (1.7)	3.0 (2.0-5.0)
Using food to calm infant fussiness (2 items, maximum score 10)	344	6.8 (1.8)	7.0 (6.0-8.0)	301	6.6 (1.7)	7.0 (5.0-8.0)
Infant satiety responsiveness score (3 items, maximum score 15)	296	7.3 (2.0)	7.0 (6.0-9.0)	262	7.3 (2.0)	7.0 (6.0-9.0)

**Table 3:** Comparison of parent feeding practice and belief outcomes at time 2 between Baby's First Food and Growing Healthy. Mean difference coefficients estimated from linear regression analysis. IQR: interquartile range.

Parent feeding practice and belief items	Distribution of outcomes						Effects of intervention	
	Baby's First Food			Growing Healthy			Mean difference (95% CI)	*P* value
	n	Mean (SD)	Median (IQR)	n	Mean (SD)	Median (IQR)		
Concerns about infant undereating or becoming underweight (4 items)	281	7.2 (2.7)	7.0 (5.0-9.0)	229	7.2 (2.8)	7.0 (5.0-9.0)	−0.14 (−0.61 to 0.34)	.57
Awareness of infant hunger and satiety cues (3 items)	281	13.2 (1.9)	14.0 (12.0-15.0)	229	12.9 (2.1)	13.0 (12.0-15.0)	−0.11 (−0.44 to 0.23)	.54
Concerns about infant overeating or becoming overweight (3 items)	281	4.5 (1.7)	4.0 (3.0-6.0)	229	4.9 (2.0)	4.0 (3.0-6.0)	0.30 (0.01 to 0.59)	.04
Feeding infant on a schedule (2 items)	282	4.9 (2.2)	5.0 (3.0-7.0)	229	5.1 (2.2)	5.0 (3.0,-7.0)	0.05 (−0.28 to 0.39)	.76
Using food to calm infant fussiness (2 items)	281	6.2 (1.9)	6.0 (5.0-8.0)	229	6.1 (1.9)	6.0 (5.0-7.0)	0.06 (−0.22 to 0.34)	.69
Infant satiety responsiveness score (3 items)	241	7.0 (2.4)	7.0 (5.0-8.0)	228	7.2 (2.2)	7.0 (6.0-8.0)	0.08 (−0.36 to 0.52)	.72

^a^Multivariate linear regression models, estimated using maximum likelihood with bootstrapped standard errors, were fitted to compare continuous outcomes between the intervention groups with adjustment for baseline parental feeding practice and belief variable, age, maternal age, maternal body mass index, whether first born, maternal country of birth, and feeding mode.

### Outcomes at Time 3

At T3, the IFQ and BEBQ satiety responsiveness scores were similar between the two groups (Table 4). Core and noncore food exposure represented by offer frequency scores and variety scores exhibited no significant between-group differences. Median core and noncore offer frequency scores were 5.0 and 0.1, respectively, indicating that infants were offed 5 core and 0.1 noncore foods per day. Similar median variety scores for core (15.0 out of a possible 22) and noncore (2.0 out of a possible 10) were also found for both groups. Of 22 core foods, the median number of foods that parent intended to offer in the future was similar between two groups (19 for Baby's First Food, 20 for Growing Healthy). Of 10 noncore foods, the median number of foods that parent intended to offer in the future was 3.0 for both Baby's First Food and Growing Healthy. No significant differences were found in the analyses of between-group differences in whether parents offer noncore foods, reoffer rejected core foods, the proportion of core and noncore foods infants had tasted and they liked, and whether the parent added salt or sugar to the infant’s foods (Table 5).

**Table 4:** Comparison of parent feeding practice and beliefs, dietary exposure, and infant food preference continuous outcomes at time 3 between Baby's First Food and Growing Healthy. Mean difference coefficients estimated from linear regression analysis. IQR: interquartile range.

Parent feeding practice and belief items	Distribution of outcomes						Effects of intervention	
	Baby's First Food			Growing Healthy			Mean difference (95% CI)	*P* value
	n	Mean (SD)	Median (IQR)	n	Mean (SD)	Median (IQR)		
Concerns about infant undereating or becoming underweight (4 items)	279	7.5 (3.0)	7.0 (5.0-9.0)	201	7.7 (3.2)	7.0 (5.0-10.0)	−0.02 (−0.59 to 0.54)	.94
Awareness of infant hunger and satiety cues (3 items)	279	13.0 (1.7)	13.0 (12.0-14.0)	201	12.6 (1.9)	13.0 (12.0-14.0)	−0.20 (−0.54 to 0.14)	.26
Concerns about infant overeating or becoming overweight (3 items)	279	4.6 (1.8)	4.0 (3.0-6.0)	202	4.7 (1.8)	4.0 (3.0-6.0)	−0.06 (−0.39 to 0.28)	.74
Feeding infant on a schedule (2 items)	279	5.4 (1.9)	6.0 (4.0-7.0)	202	5.5 (1.9)	5.0 (4.0-7.0)	−0.10 (−0.41 to 0.21)	.54
Using food to calm infant fussiness (2 items)	279	5.8 (1.9)	6.0 (4.0-7.0)	201	5.7 (1.8)	6.0 (4.0-7.0)	0.06 (−0.24 to 0.37)	.69
Infant satiety responsiveness score (3 items)	250	7.2 (2.1)	7.0 (6.0-8.0)	181	6.8 (2.1)	7.0 (6.0-8.0)	−0.21 (−0.62 to 0.19)	.30
Core food offer frequency score	276	5.2 (2.0)	5.0 (3.8-6.4)	202	5.1 (1.9)	5.0 (3.7-6.4)	−0.07 (−0.44 to 0.30)	.70
Non-core offer food frequency score	275	0.3 (0.5)	0.1 (0.0-0.5)	202	0.3 (0.4)	0.1 (0.0, 0.4)	−0.06 (−0.15 to 0.03)	.19
Core food variety score (0-22)	276	14.8 (3.1)	15.0 (13.0-17.0)	202	15.0 (3.0)	15.0 (13.0-17.0)	0.18 (−0.40 to 0.76)	.55
Non-core food variety score (0-10)	275	2.6 (2.0)	2.0 (1.0-4.0)	202	2.5 (2.2)	2.0 (1.0-4.0)	0.03 (−0.34 to 0.41)	.87
Number of core foods parent will offer again (0-22)	277	18.7 (2.7)	19.0 (17.0-21.0)	202	19.1 (2.4)	20.0 (18.0-21.0)	0.39 (−0.10 to 0.88)	.12
Number of non-core foods parent will offer again (0-10)	277	3.2 (2.3)	3.0 (1.0-5.0)	202	3.2 (2.5)	3.0 (1.0-5.0)	0.00 (−0.43 to 0.44)	.99

^a^Multivariate linear regression models, estimated using maximum likelihood with bootstrapped standard errors, were fitted to compare continuous outcomes between the intervention groups with adjustment for baseline parental feeding practice and beliefs variable, age, maternal age, maternal body mass index, whether first born, maternal country of birth, and feeding method.

**Table 5:** Comparison of parent feeding practice and belief binary outcomes at time 3 between Baby's First Food and Growing Healthy.

Parent feeding practice and belief items		Baby's First Food, n (%)	Growing Healthy, n (%)	Effects of intervention	
				Odds ratio (95% CI)	*P* value
**Did parent offer infant any noncore foods**				0.96 (0.59 to 1.55)	.81
	No	54 (19.6)	44 (21.8)		
	Yes	221 (80.4)	158 (78.2)		
**Proportion of core foods infant has tasted that they liked**				1.22 (0.83 to 1.80)	.36
	Not all	162 (58.3)	111 (54.7)		
	All	116 (41.7)	92 (45.3)		
**Proportion of noncore foods infant has tasted that they liked**				1.27 (0.67 to 2.41)	.46
	Not all	35 (15.3)	19 (11.9)		
	All	194 (84.7)	140 (88.1)		
**Proportion of disliked core foods the parent intended to offer again**				1.17 (0.64 to 2.14)	.61
	Not all	42 (25.9)	26 (23.4)		
	All	120 (74.1)	85 (76.6)		
**Does parent add salt or sugar to foods infant eats**				0.82 (0.48 to 1.41)	.47
	Never	235 (84.8)	174 (86.1)		
	Some of the time	42 (15.2)	28 (13.9)		

## Discussion

### Principal Findings

This study considered the effects of an mHealth intervention on parental feeding practices and cognitions, infants’ food preferences, and infants’ satiety responsiveness. In this study, we noted very few differences between the intervention and comparison groups in the measured outcomes, suggesting that although mHealth offers many advantages to both the researcher and participant over traditional approaches, further evidence on the most effective approaches for achieving and measuring outcomes in infant feeding are needed.

At each of the time points, just one of the tested relationships (concern about infant overeating or becoming overweight at T2) differed between the two groups. The majority of other studies using traditional intervention approaches (face-to-face) have been able to support parents to use some desirable infant feeding practices and eating outcomes for children, but not all. The NOURISH trial, for instance, which recruited first-time mothers and their 4- to 7-month old infants to a responsive feeding intervention, was able to influence infant satiety responsiveness [50] and only one of the IFQ subscales at 14 months [51]. In the Melbourne InFANT Program, a cluster randomized controlled trial (RCT) delivering diet and feeding education to first-time Australian mothers, differences in intervention and control group parents’ use of *food as a reward* were observed, but no changes were seen for the other measured feeding practices at 18 months of age [52]. The POI.nz RCT also noted group differences in *pressure to eat* along with *child control* and *encourage nutrient-dense foods* but none of the other feeding practices after the provision of information and support on feeding and food [53]. The SLIMTIME study also showed effects on repeated exposure to vegetables, as well as the number of feeds per day, but the latter only for breastfed infants when a responsive feeding intervention was administered to first-time American mothers [54]. The reasons underlying the effects of face-to-face multicomponent interventions on some feeding practices are unclear because of their heterogeneous designs and complex nature [55]. However, a recent intervention with slightly older children noted the important influence of children’s innate individual characteristics (eg, temperament) on intervention effectiveness [56], and it is possible that further tailoring of intervention to these particular characteristics would enhance effectiveness.

In relation to the findings on food exposure, infants in the intervention group were equally likely as those in the comparison group to be exposed to and like core (as well as noncore) foods and were equally likely to be reoffered those foods that they initially disliked. The Growing Healthy program aimed to encourage parents to repeatedly expose their infants to core foods, even when initially rejected, and to avoid exposure to noncore foods. This could be a result of selection bias: it appeared that parents in both groups had similar intentions and behaviors, and in many (though not all) instances, these were close to ideal. For this reason, showing an effect of the intervention was challenging with the available sample size and measurement tools. Less ideal feeding practices may also only emerge later when children present more behavioral challenges such a food neophobia or fussy eating at older ages [57,58], along with increasing independence and assertiveness such as in toddlerhood and beyond [59]. Furthermore, interventions targeting children or infants at high risk of (further) excess weight gain are likely to be more effective in producing change. Lioret et al [60], for instance, noted that changes in diet quality were only associated with reduced z-score BMI for school children who were already overweight at baseline. In this study, it is possible that a subset of parents with higher risk behaviors existed, perhaps the approximately one-quarter of parents who did not intend to offer a disliked core food again, or the 14% to 15% who were adding salt or sugar to foods their infant ate at least some of the time.

The influence of the unique design features of the intervention on participants’ behaviors may have also affected results. Health behavior programs delivered by mHealth are likely to be more effective when they meet several criteria including that they have a theoretical basis, suitable BCTs are employed over a suitable period of time, the content is appropriate, and the design characteristics and mode of delivery of the intervention are appealing to the participants [61,62], although many gaps exist in our understanding of effective design features for different behaviors and contexts [61]. Indeed the most effective features for child obesity prevention interventions (mHealth or otherwise) is still unclear [63]. It is also important that the program be tailored to the individual needs of the participants [61]. The Growing Healthy program was designed with these criteria in mind [40], and feedback from participants suggested that in many aspects, the program was able to meet participants’ expectations and needs [43]. Retention across the study time frame was also high (80%), suggesting that there were benefits for participants in continuing to participate. However, preferred design and delivery characteristics differ across individuals and with different health behaviors. It could be that further tailoring of the intervention content to other characteristics of parent-infant dyads, such as their current knowledge around infant feeding, whether their child was a particularly avid eater or born at a high or low birth weight, or child temperament, may improve future studies. In Growing Healthy, a number of feeding behaviors were targeted (eg, breastfeeding, formula feeding, food exposure, and feeding to appetite) across a diverse sample of participants, and designing an intervention in such a way that it can optimally meet the diverse needs of all participants for all target behaviors is a challenge.

Another important consideration in explaining the effects reported here relates to the dose of the intervention. Although many of the challenges faced in an mHealth intervention of healthy infant feeding are similar to those faced in other parent feeding interventions (eg, effective BCTs and measurement of outcomes), there are some unique challenges associated with the mHealth delivery mode that may have affected the results. For instance, there were indications that improvements could be made to the delivery of the program: technical problems related to operating system upgrades that saw the app temporarily cease functioning likely affected the dose of the intervention received by some of the participants and consequently, may have reduced potential impact of the intervention. This may have affected participant engagement. Participant engagement describes how often participants accessed various elements of the app, read push notifications, participated in the forum, looked at the website, and over what period of time [64]. Engagement with mHealth programs is typically high upon joining the program and diminishes thereafter, and app analytics indicated that participants in Growing Healthy followed this pattern [43,64]. However, although overall engagement declined with time, the sections of the program covering topics related to feeding and sleeping were among the most accessed by participants, suggesting that parents were interested in the topics and sought information regarding these constructs. It is unclear, then, whether the dose of the intervention received was lower than that needed to produce detectable changes in behaviors. A larger sample that enabled subgroup analyses based on engagement and interactions with particular design features may have provided some explanation.

The Growing Healthy program drew on the BCW framework and, as part of this, the Capability, Opportunity, Motivation, and Behavior (COM-B) framework to select BCTs likely to be effective in changing the target behaviors [42]. Available evidence on the likely relationships between the COM-B elements and the BCTs most likely to be effective in changing them was used; however, the effects of the many possible BCTs (eg, providing information about possible consequences, modeling [65], and their delivery [eg, videos and text]) on infant feeding have not previously been tested for their independent effects on COM-B, especially in an mHealth context. As such, it is difficult to know how successful each of these were in influencing target behaviors. Qualitative feedback from participants suggested that the program influenced some of the antecedents of behaviors (capability and opportunity in particular) [43]; however, the effects of these were not detected in the outcome measures. Interventions adhering to behavioral theories such as the BCW and who use particular BCTs are more likely to be effective than those that do not [66,67], although further work is needed to identify which components of such theories and which particular BCTs are likely to produce sustainable outcomes for different health behaviors in a range of contexts. It is possible, for instance, that alternative determinants of behaviors such as social norms could have had an influence on outcomes, and these were not addressed here but could be in the future with appropriate BCTs.

An additional challenge, common to all studies of parental feeding, is the effective measurement of parental feeding practices. In this study, the IFQ was used to measure parental infant feeding practices. At the time of designing the study, this was the only available self-reported measure of relevant parent infant feeding practices (to the authors’ knowledge). The IFQ was developed with infants from 6 months of age. In this study though, parents were initially asked to complete it when their infant was aged less than 3 months, and it is not known how appropriate the IFQ is with younger infants. Furthermore, the IFQ has not been validated against observational measures of parental feeding, or against objective measures of infant’s eating and weight, and therefore its capacity to accurately reflect parent’s feeding practices and beliefs is unknown. Additionally, the measure of infant food liking and parental intentions to reoffer foods were purpose-developed for this study and has not been validated, although it is possible that parent measures of their infant feeding practices and their infant’s food preferences are affected by social desirability and recall bias. Greater attention to validating measures of parent feeding practices and infant food preferences is urgently needed if the effects of interventions are to be confidently demonstrated.

A final but important consideration in interpretation of the results is that this study used a quasi-experimental design, and as such, the participants were not randomly assigned. Consequently, the two groups differed at baseline in some infant (eg, age) and parent (eg, parity) characteristics (although were similar in the majority of key criteria). The analyses took account of these differences; however, adequately powered RCTs are needed to confidently demonstrate effects.

### Conclusions

Although mHealth can be effective in promoting health behaviors and offers many advantages in health promotion, the results of this study suggest that design and delivery characteristics needed to maximize the impact of mHealth interventions on infant feeding are uncertain. Further tailoring of content, including BCTs, to individual circumstances and characteristics may improve efficacy in different contexts. Furthermore, improved measures of outcomes, including those that are objectively measured or sensitive enough to reveal small changes in behaviors that are likely achieved by low-dose mHealth interventions, may be needed if the effects of mHealth interventions are to be detected.

## Appendix

Multimedia Appendix 1 Items under each parental feeding practice and belief outcome and infant satiety responsive score and their Cronbach alpha.

## Abbreviations

BCT: behavior change technique
BCW: Behaviour Change Wheel
BEBQ: Baby Eating Behaviour Questionnaire
BMI: body mass index
COM-B: Capability Opportunity Motivation and Behavior
IFQ: Infant Feeding Questionnaire
IQR: interquartile range
mHealth: mobile health
RCT: randomized controlled trial
T1, T2, T3: baseline, time 2, time 3
