# Supplementary material for: Impact of the Growing Healthy mHealth Program on Maternal Feeding Practices, Infant Food Preferences, and Satiety Responsiveness: Quasi-Experimental Study
Source: JMIR Mhealth Uhealth. 2018 Apr 25;6(4):e77. doi: 10.2196/mhealth.9303 (PMC5943630; doi:10.2196/mhealth.9303)
Supplement: Multimedia Appendix 1 [file mhealth_v6i4e77_app1.pdf]

Supplementary Table 1. Items under each parental feeding practice and belief outcome and infant satiety responsive score and their Cronbach alpha.

Factor and questions		Response	Cronbach alpha		
			Baseline	T2	T3
**Concerns about infant undereating or becoming underweight (4 items)**			.67	.70	.78
	Do you worry that your baby is not eating enough?	1=never, 2=rarely, 3=sometimes, 4=mostly, 5=always			
	Is it a struggle to get your baby to eat or feed?	1=never, 2=rarely, 3=sometimes, 4=mostly, 5=always			
	I am worried that my baby will become underweight	1=never, 2=rarely, 3=sometimes, 4=mostly, 5=always			
	If I did not encourage my baby to eat or feed, then they would not eat or feed enough.	1=never, 2=rarely, 3=sometimes, 4=mostly, 5=always			
**Awareness of infant hanger and satiety cues (3 items)**			.66	.71	.59
	My baby knows when they are hungry	1=disagree a lot, 2=disagree a little, 3=no strong feelings either way, 4=agree a little, 5=agree a lot			
	My baby knows when they are full	1=disagree a lot, 2=disagree a little, 3=no strong feelings either way, 4=agree a little, 5=agree a lot			
	I know when my baby is full	1=disagree a lot, 2=disagree a little, 3=no			

		strong feelings either way, 4=agree a little, 5=agree a lot			
**Concerns about infant overeating or becoming overweight (3 items)**				.57	.53
	Do you worry that your baby is eating or feeding too much?	1=disagree a lot, 2=disagree a little, 3=no strong feelings either way, 4=agree a little, 5=agree a lot			
	Do you get upset if your baby eats or feeds too much?	1=disagree a lot, 2=disagree a little, 3=no strong feelings either way, 4=agree a little, 5=agree a lot			
	I am worried that my baby will become overweight	1=disagree a lot, 2=disagree a little, 3=no strong feelings either way, 4=agree a little, 5=agree a lot			
**Feeding infant on a schedule (2 items)**			.68	.77	.68
	Do you let your baby eat or feed whenever they want to?	1=never, 2=rarely, 3=sometimes, 4=mostly, 5=always			
	Do you feed your baby at set times?	1=never, 2=rarely, 3=sometimes, 4=mostly, 5=always			
**Using food to calm infant fussiness (2 items)**				.75	.69
	When your baby gets unsettled, is feeding them the first thing you do?	1=never, 2=rarely, 3=sometimes, 4=mostly, 5=always			
	Feeding my baby is the best way to stop them being unsettled	1=disagree a lot, 2=disagree a little, 3=no strong feelings either way, 4=agree a little, 5=agree a			

		lot			
**Infant satiety responsiveness score (3 items)**<br><br>(these items also had a ‘don’t know’ response option, which was subsequently coded as missing)			.61	.74	.75
	My baby gets full before taking all of the milk or food I thought they should have	1=never, 2=rarely, 3=sometimes, 4=mostly, 5=always			
	My baby finds it difficult to manage a complete feed	1=never, 2=rarely, 3=sometimes, 4=mostly, 5=always			
	My baby gets full up easily	1=never, 2=rarely, 3=sometimes, 4=mostly, 5=always			
